# Statewide Medicaid Expansion and Survival in Resectable Non–Small Cell Lung Cancer

**DOI:** 10.1001/jamanetworkopen.2025.45996

**Published:** 2025-12-01

**Authors:** Rohin Gawdi, Shahidul Islam, Calista Sha, Shangyi Liu, Lawrence R. Glassman, Kevin M. Hyman, Julissa E. Jurado, David Zeltsman, Paul C. Lee

**Affiliations:** 1 Department of Surgery, Donald and Barbara Zucker School of Medicine at Hofstra/Northwell, Manhasset, New York; 2 Division of Thoracic Surgery, Massachusetts General Hospital, Boston; 3 Biostatistics Unit, Office of Academic Affairs, Northwell Health, New Hyde Park, New York; 4 Department of Cardiovascular and Thoracic Surgery, Northwell Health Long Island Jewish Medical Center, New Hyde Park, New York

## Abstract

**Importance:**

Medicaid expansion under the Affordable Care Act broadened eligibility for government-funded health coverage. This may have improved access to early diagnosis and rapid treatment, potentially impacting survival in patients with resectable non–small cell lung cancer (NSCLC), a leading cause of cancer-related death in the US.

**Objective:**

To evaluate the association between state-level Medicaid expansion and all-cause mortality in patients with resectable NSCLC.

**Design, Setting, and Participants:**

This cohort study of patients aged 20 to 64 years who were diagnosed with stage I to IIIA NSCLC used data from the Surveillance, Epidemiology, and End Results registry collected between January 1, 2006, to December 31, 2019, with outcomes administratively censored at 2 and 4 years. Data were analyzed between March 1 and September 10, 2025.

**Exposure:**

Medicaid expansion status of patients’ state of residence at diagnosis. Patients’ states of residence were categorized as nonexpansion, early expansion (2011), 2014 expansion, or late expansion (after 2014).

**Main Outcomes and Measures:**

Primary outcomes were 2- and 4-year all-cause mortality. Cox proportional hazards regression models within a difference-in-differences framework were used to estimate adjusted hazard ratios (HRs) for death. Secondary analyses evaluated postexpansion mortality trends and changes in early-stage (I-II) diagnoses using logistic regression.

**Results:**

Among 53 842 patients included in the analysis (24 849 [46.2%] 60-64 years of age; 27 027 [50.2%] male), propensity score–matched analyses showed lower 2-year mortality in states with early expansion (HR, 0.95; 95% CI, 0.91-0.99; *P* = .02) and 2014 expansion (HR, 0.91; 95% CI, 0.86-0.95; *P* < .001) compared with nonexpansion control states, whereas no significant difference was observed in late expansion states (HR, 0.95; 95% CI, 0.89-1.02; *P* = .15). All expansion groups showed decreased mortality. Mortality decreases were observed after the first 3 years and persisted throughout the study period. The proportion of early-stage diagnoses did not change post expansion, suggesting benefits may have been mediated by improved postdiagnostic care rather than earlier detection.

**Conclusions and Relevance:**

In this cohort study of patients with stage I to IIIA NSCLC, Medicaid expansion was associated with lower mortality at 2 and 4 years. These findings suggest that expanded insurance coverage may have enhanced access to effective cancer care and improved population-level outcomes.

## Key Points

**Question:**

Did expanded Medicaid eligibility under the Affordable Care Act improve survival for patients with resectable non–small cell lung cancer (NSCLC)?

**Findings:**

In this cohort study of 53 842 patients with NSCLC, statewide Medicaid expansion status was associated with increased 2-year survival in states that expanded coverage in 2014 or earlier and increased 4-year survival for all Medicaid expansion groups. A time lag for mortality differences was observed after policy implementation, but there was no corresponding increase in early-stage diagnoses after Medicaid expansion.

**Meaning:**

These findings suggest that Medicaid expansion was associated with decreased mortality hazards for patients diagnosed with resectable NSCLC, underscoring the critical role health policy plays in cancer outcomes.

## Teaser

This cohort study investigates associations between state-level Medicaid expansion and survival outcomes among US patients younger than 65 years with stage I to IIIA non–small cell lung cancer.

## Introduction

Lung cancer remains the leading cause of cancer-related death in the US, with non–small cell lung cancer (NSCLC) accounting for approximately 85% of cases.^1^ Among patients with resectable NSCLC, early detection and timely access to multimodal therapy are critical determinants of long-term survival.^2,3,4,5^ However, for low-income and underinsured patients, life-prolonging interventions are often delayed or inaccessible due to socioeconomic barriers.^5,6^ The Affordable Care Act (ACA), implemented in 2014, sought to reduce this disparity by broadening Medicaid eligibility to all adults with incomes below 138% of the federal poverty level and providing states with matching federal funding.^7^ State participation in this program is optional,^8,9^ thus Medicaid expansion time frames differ across states. This provides a natural opportunity to evaluate the clinical consequences of Medicaid expansion on lung cancer outcomes.

Several studies have shown that expanded Medicaid eligibility is associated with cancer diagnosis at earlier stages,^10,11,12,13,14^ higher rates of treatment,^15,16,17,18^ and improved short-term survival in underserved populations.^19,20,21^ However, the long-term policy effects of Medicaid expansion on patients with potentially resectable NSCLC—who stand to benefit most from early and comprehensive treatment—remains unclear. Prior work^19,20,21^ has been limited by short follow-up periods, narrow policy criteria, or inability to account for concurrent secular trends. To address these gaps, we designed a cohort study to investigate associations between state-level Medicaid expansion and survival outcomes among patients younger than 65 years with stage I to IIIA NSCLC. Resectable NSCLC serves as an ideal policy sentinel for Medicaid expansion because it is a high-mortality, high-cost disease in which timely diagnosis and multifaceted treatment directly determine survival.^3^ The disproportionate burden among low-income, rural, working-class, and publicly insured populations makes NSCLC a powerful model to evaluate the clinical outcomes of Medicaid expansion among disadvantaged groups and the population at large.^22,23,24,25^

## Methods

### Study Design and Population

We conducted an observational cohort study using generalized difference-in-differences (DID) models to examine associations between state-level Medicaid expansion and all-cause mortality among patients with stage I to IIIA NSCLC. We queried diagnoses among patients aged 20 to 64 years (January 1, 2006, to December 31, 2019) from the Surveillance, Epidemiology, and End Results (SEER) Research Plus database, version 8.4.4, which provided granular county-level data unavailable in the standard database.^26^ We excluded patients older than 65 years (ie, Medicare-eligible patients) and those with incomplete data. No imputation was performed. Registry linkage ensured no loss to follow-up beyond administrative censoring. Approval was obtained from the Institutional Review Board of Northwell Health with a waiver of consent for use of deidentified data. We used the Strengthening the Reporting of Observational Studies in Epidemiology (STROBE) guidelines to ensure transparency in reporting study design, setting, participants, exposures, outcomes, statistical methods, results, and limitations.

### Defining Resectable Disease

Because SEER’s treatment fields are inconsistently reported, we defined resectable NSCLC by stage (a standardized proxy for patients amenable to surgical management). Patients coded as having stage III NSCLC, not otherwise specified, were excluded.

### Exposure

The primary exposure variable was state-level Medicaid expansion status at the time of diagnosis, categorized by each state’s adoption timeline of the ACA Medicaid expansion provision: nonexpansion (Utah and Georgia), early expansion (California, Connecticut, New Jersey, and Washington [effective January 2011]), on-time expansion (Hawaii, Kentucky, Iowa, and New Mexico [effective January 2014]), and late expansion (Alaska and Louisiana).^27^ We defined 4 time periods to reflect pre-expansion and postexpansion eras: 2006 to 2010 (pre-expansion), 2011 to 2013 (early expansion), 2014 to 2016, and 2017 to 2019 (late expansion). Across analyses, patients in nonexpansion states served as controls.

### Covariates

Covariates included patient age, sex, race and ethnicity, cancer stage, and county rurality (2010 Rural-Urban Continuum Codes),^28^ selected based on SEER availability and known associations with NSCLC outcomes.^29,30,31,32^ Race and ethnicity data were abstracted from registry coding rather than self-report and were categorized as Hispanic (all races); non-Hispanic American Indian or Alaska Native; non-Hispanic Asian, Native Hawaiian, or Other Pacific Islander; non-Hispanic Black; non-Hispanic White; and unknown race. We included race and ethnicity not as a biological determinant but rather as a proxy for exposure to structural inequities in health care. The purpose of this adjustment was to account for baseline disparities that might confound associations between Medicaid expansion and survival. Stage at diagnosis was included as a matching covariate to help estimate the association between Medicaid expansion and mortality conditional on resectability, rather than through changes in stage distribution. This approach helps us capture whether Medicaid expansion improves outcomes through enhanced postdiagnosis care, such as timely treatment and care coordination. We independently evaluated early-stage diagnosis trends in separate models to avoid conflating these distinct mechanisms.

### Propensity Score Matching

Propensity scores were estimated separately for each expansion group against the control group using logistic regression with sociodemographic and clinical covariates. The logit of estimated scores was calculated and used as the matching metric. We performed 1:1 nearest-neighbor matching using the MatchIt R package,^33^ with caliper widths of 0.2 SDs of the logit scale to optimize match quality. We plotted propensity score distributions and standardized mean covariate differences to ensure matching quality.

### Outcome

The primary outcome was all-cause mortality within 2 and 4 years of diagnosis. Patients were censored at the date of last known follow-up if no death was recorded, ensuring that censoring and death were not misclassified.

### Statistical Analysis

All analyses were conducted in R, version 4.4.1 (R Program for Statistical Computing) between March 1 and September 10, 2025.^34^ We compared baseline characteristics using χ^2^ testing for categorical variables. Survival probabilities within each era were generated with Kaplan-Meier methods; log-rank testing and pairwise comparisons were performed across groups.^35^ Statistical significance was defined as a 2-sided *P* < .05. Benjamini-Hochberg procedures were applied to control for false discovery rates from multiple pairwise comparisons.

We conducted generalized DID analyses using Cox proportional hazards regression models with interaction terms between time period and expansion status to estimate mortality hazard ratios (HRs) and 95% CIs. DID Cox proportional hazards regression models were applied to both unmatched and matched cohorts. All models incorporated robust SEs clustered at the state level to partially control for unmeasured state-level characteristics and regional policy environments. DID analyses compared overall survival of each expansion group against the control before and after Medicaid expansion: early expansion (2006-2010 vs 2011-2019), 2014 expansion (2006-2013 vs 2014-2019), and late expansion (2006-2016 vs 2017-2019). To assess associations with policy over time, we subdivided postexpansion periods into implementation (first 3 years) and postimplementation (beyond the third year) eras. Separate DID models were constructed for each era and survival interval as follow-up permitted. Analysis of 5-year mortality (censoring at 60 months) was conducted as a sensitivity analysis; however, this approach underrepresented patients diagnosed in the later expansion years due to insufficient follow-up time, potentially biasing against detecting longer-term benefits of expansion.

#### Parallel Trends Assumption and Falsification Analyses

A critical aspect of the validity of DID models is the parallel trends assumption, which posits that existing trends would remain consistent across treatment and control groups in absence of policy changes. We assessed this assumption using placebo-falsification models and plots of mortality and early-stage diagnosis.

#### Secondary and Sensitivity Analyses

To assess whether mortality changes were associated with earlier detection, we analyzed patterns in early-stage diagnosis across the entire cohort. The unit of analysis was individual patients, and outcomes were binary (stage I plus II vs stage IIIA). Models estimated odds of early-stage diagnosis using logistic regression with DID interaction terms between expansion status and time, incorporating state-level clustered robust SEs; odds ratios (ORs) and 95% CIs were reported.

Exploratory multivariable Cox proportional hazards regression models within the propensity score–matched cohort identified demographic and clinical characteristics associated with mortality risk. A similar model, using interaction terms between patient characteristics and time period, was made to assess differential associations of Medicaid expansion with survival across sociodemographic groups. These models controlled for age, sex, marital status, race, county income quintile, and rurality; interaction terms quantified preexisting associations with survival, and how those disparities changed after expansion. To validate our Cox proportional hazards regression models, we tested the proportional hazards assumption and conducted sensitivity analyses using time-treatment interaction terms and Royston-Parmar models.

## Results

Our cohort included 53 954 patients, of whom 112 were excluded due to incomplete sociodemographic or outcomes data (Figure). Of the included 53 842 patients, 26 815 (49.8%) were female and 27 027 (50.2%) were male; 13 046 (24.2%) were 54 years or younger; 15 947 (29.6%) were 55 to 59 years of age; and 24 849 (46.2%) were 60 to 64 years of age. A total of 2968 patients (5.5%) were Hispanic; 258 (0.5%), non-Hispanic American Indian or Alaska Native; 3334 (6.2%), non-Hispanic Asian, Native Hawaiian, or Other Pacific Islander; 7457 (13.8%), non-Hispanic Black; 39 723 (73.8%), non-Hispanic White; and 102 (0.2%), unknown race. Because outcomes were ascertained through registry linkage, there was no loss to follow-up beyond administrative censoring at last known follow-up. Patients were grouped into nonexpansion (n = 9896), early expansion (n = 28 825), 2014 expansion (n = 10 442), and late expansion (n = 4679) cohorts (eFigure 1 and eTable 1 in Supplement 1). Baseline sociodemographic and treatment characteristics differed significantly across groups (eTable 2 in Supplement 1).

**Figure.**
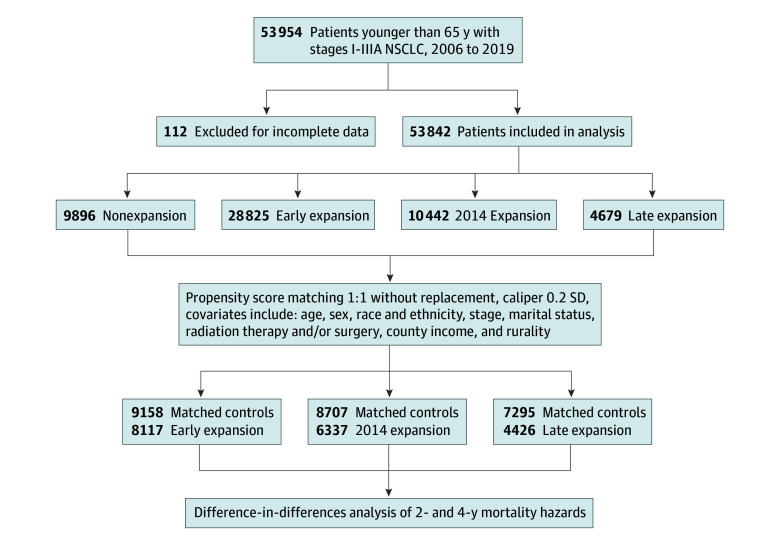
Schematic of Study Population Selection Stepwise inclusion and exclusion criteria used to derive the final analytic cohort from the Surveillance, Epidemiology, and End Results database. Patients in the experimental and control groups for each comparison were filtered out if the logit of the propensity score exceeded 0.2 SD on the logit scale. NSCLC indicates non–small cell lung cancer.

In whole-population DID models (Table 1), the 2014 expansion group experienced a significant decrease in postexpansion mortality (HR for DID interaction, 0.94; 95% CI, 0.90-0.98; *P* = .005), as did the late expansion group (HR for DID interaction, 0.93; 95% CI, 0.87-0.99; *P* = .02). While the early expansion group showed no significant decrease in unmatched models (HR for DID interaction, 0.97; 95% CI, 0.94-1.01; *P* = .10), postmatching DID models demonstrated a significant mortality reduction relative to nonexpansion states (HR for DID interaction, 0.95; 95% CI, 0.91-0.99; *P* = .02). The 2014 expansion group similarly showed a significant decrease in mortality (HR for DID interaction, 0.91; 95% CI, 0.86-0.95; *P* < .001); however, the late expansion group’s patterns of relative survival increase were no longer significant (HR for DID interaction, 0.95; 95% CI, 0.89-1.02; *P* = .15). Kaplan-Meier curves for the whole cohort with log-rank and pairwise comparisons are shown in eFigure 2 and eTable 3 in Supplement 1. Upon propensity score matching, covariate balance (mean imbalance <0.15) was achieved across all comparisons (eFigures 3 and 4 in Supplement 1).

**Table 1.** Association Between Medicaid Expansion Status and 2-Year Overall Survival in Patients With Resectable Non–Small Cell Lung Cancer Before and After Propensity Score Matching

Group	Whole population		Propensity score–matched cohorts^a^	
	HR (95% CI)	*P* value	HR (95% CI)	*P* value
Nonexpansion	1 [Reference]	NA	1 [Reference]	NA
Early expansion	0.97 (0.94-1.01)	.10	0.95 (0.91-0.99)	.02
2014 expansion	0.94 (0.90-0.98)	.005	0.91 (0.86-0.95)	<.001
Late expansion	0.93 (0.87-0.99)	.02	0.95 (0.89-1.02)	.15

Abbreviations: HR, hazard ratio; NA, not applicable.

^a^ Propensity score matching was performed using Surveillance, Epidemiology, and End Results variables for age, sex, race and ethnicity, county-level income, and stage at diagnosis. Expansion groups were compared with nonexpansion states during matched time periods: early expansion, 2006 to 2010 vs 2011 to 2019; 2014 expansion, 2006 to 2013 vs 2014 to 2019; and late expansion, 2006 to 2016 vs 2017 to 2019. Race and ethnicity were included as a proxy for structural inequities in health care rather than as biological determinants of outcome.

Era-stratified DID analyses (Table 2) assessed mortality across successive postexpansion eras. For early expansion states, no significant survival changes were observed during the 3-year (2011-2014) implementation window (2-year HR, 1.07 [95% CI, 0.97-1.17; *P* = .17]; 4-year HR, 1.07 [95% CI, 0.99-1.14; *P* = .06]). Decreased mortality hazards were seen in the 2014-2019 postimplementation period (2-year HR, 0.89 [95% CI, 0.85-0.93; *P* < .001]; 4-year HR, 0.91 [95% CI, 0.89-0.94; *P* < .001]) and across the overall 2011-2019 postexpansion period (2-year HR, 0.95 [95% CI, 0.91-0.99; *P* = .02]; 4-year HR, 0.97 [95% CI, 0.94-0.99; *P* = .03]). Similarly for the 2014 expansion group, no differential survival change was observed in the first 3 years (2014-2016) after expansion (2-year HR, 0.95 [95% CI, 0.88-1.03; *P* = .25]; 4-year HR, 0.97 [95% CI, 0.95-0.99; *P* = .004]). A significant mortality decrease then became evident in the postimplementation (2017-2019) era (2-year HR, 0.85 [95% CI, 0.77-0.94; *P* = .001]; 4-year HR, 0.86 [95% CI, 0.80-0.93; *P* < .001]), and was maintained during the entire postexpansion (2014-2019) period (2-year HR, 0.91 [95% CI, 0.86-0.95; *P* < .001]; 4-year HR, 0.92 [95% CI, 0.89-0.95; *P* < .001]). For late expansion states, no significant 2-year mortality decrease was detected during implementation (2017-2019; HR, 0.95; 95% CI, 0.89-1.02; *P* = .15), although a significant 4-year survival increase was observed (HR, 0.92; 95% CI, 0.89-0.94; *P* < .001). Postimplementation analysis for late expansion was limited by the lack of follow-up data available in SEER beyond 2021. Propensity score–matched Kaplan-Meier curves with log-rank and pairwise comparisons can be seen in eFigure 5 and eTable 4 in Supplement 1. Placebo-falsification analyses showed no significant DID placebo effect on 2-year survival (β = –0.06 [SE, 0.34]; *P* = .86), indicating no differential pre-expansion survival trends (eTable 5 and eFigures 6 and 7 in Supplement 1). Adjusted 2-, 4-, and 5-year mortality hazards and survival likelihoods are shown in eTables 6 and 7 in Supplement 1.

**Table 2.** Generalized Difference-in-Differences Analysis of 2-Year and 4-Year Non–Small Cell Lung Cancer Survival After Medicaid Expansion^a^

Medicaid expansion era	2-y Survival			4-y Survival		
	HR (95% CI)	*P* value	HR (95% CI)		*P* value
Early expansion					
Pre-expansion (2006-2010)	1 [Reference]	NA	1 [Reference]		NA
Implementation (2011-2013)	1.07 (0.97-1.17)	.17	1.07 (0.99-1.14)		.06
After implementation (2014-2019)	0.89 (0.85-0.93)	<.001	0.91 (0.89-0.94)		<.001
Total postexpansion	0.95 (0.91-0.99)	.02	0.97 (0.94-0.99)		.03
2014 expansion					
Pre-expansion (2006-2013)	1 [Reference]	NA	1 [Reference]		NA
Implementation (2014-2016)	0.95 (0.88-1.03)	.25	0.97 (0.95-0.99)		.004
After implementation (2017-2019)	0.85 (0.77-0.94)	.001	0.86 (0.80-0.93)		<.001
Total postexpansion	0.91 (0.86-0.95)	<.001	0.92 (0.89-0.95)		<.001
Late expansion					
Pre-expansion (2006-2016)	1 [Reference]	NA	1 [Reference]		NA
Implementation (2017-2019)	0.95 (0.89-1.02)	.15	0.92 (0.89-0.94)		<.001

Abbreviations: HR, hazard ratio; NA, not applicable.

^a^ Analyses were conducted using propensity score–matched populations and adjusted for demographic, clinical, and socioeconomic covariates. The early postexpansion period (first 3 years) is the implementation era, while the remaining years until the end of follow-up comprise the postimplementation era. Reported HRs reflect the estimated interaction effect of Medicaid expansion on mortality in each era, with corresponding 95% CIs and *P* values. HR of less than 1 refers to reduced mortality hazard.

In the propensity score–matched cohort, exploratory multivariable Cox proportional hazards regression identified several demographic and clinical factors independently associated with 2-year mortality (eTable 8 in Supplement 1). Age younger than 55 years (HR, 0.87; 95% CI, 0.79-0.95; *P* = .001), female sex (HR, 0.69; 95% CI, 0.65-0.71; *P* < .001), non-Hispanic Asian, Native Hawaiian, or Other Pacific Islander race (HR, 0.49; 95% CI, 0.32-0.77; *P* = .002), and married or domestic partnership status (HR, 0.76; 955 CI, 0.71-0.80; *P* < .001) were associated with improved mortality. Conversely, residing in smaller metropolitan (HR, 1.21; 95% CI, 1.04-1.41; *P* = .01) or rural (HR, 1.23;95% CI, 1.08-1.39; *P* < .001) counties was associated with higher mortality. No specific racial or ethnic minority group showed a significant difference in mortality compared with non-Hispanic White patients. Interaction models assessed differential benefit from Medicaid expansion across sociodemographic groups (eTable 9 in Supplement 1). We observed no relative benefit by sex, racial subgroup, or county rurality. Compared with single individuals, improved survival was observed for married individuals and those with domestic partners (HR, 0.91; 95% CI, 0.84-0.99; *P* = .04) and previously married individuals (HR, 0.90; 95% CI, 0.81-0.99; *P* = .03). Conversely, patients residing in the counties with the lowest income quintiles experienced higher hazards of mortality from before to after Medicaid expansion compared with counties with the highest income quintile (quintile 1 vs 5 HR, 1.19 [95% CI, 1.03-1.38; *P* = .02]; quintile 2 vs 5 HR, 1.16 [95% CI, 1.03-1.32; *P* = .02]).

We then examined the impact of Medicaid expansion on early-stage (stage I and II) NSCLC diagnosis (Table 3). A small, nonsignificant DID interaction (interaction term OR, 1.07; 95% CI, 0.97-1.17; *P* = .18) suggested Medicaid expansion was not associated with meaningful shifts in early-stage diagnosis. While patients in expansion states had overall higher odds of stage I and II diagnoses (OR, 1.12; 95% CI, 1.05-1.18; *P* < .001), early-stage diagnoses decreased over the study time frame (OR, 0.91; 95% CI, 0.84-0.99; *P* = .02). Placebo-falsification modeling for early-stage diagnosis showed no significant difference in trends between groups (β = –0.005 [SE, 0.06]; *P* = .94), consistent with parallel trends (eTable 10 in Supplement 1). Time-treatment interaction (eTable 11 in Supplement 1) and Royston-Parmar parametric model (eTable 12 in Supplement 1) results validated the Cox proportional hazards regression models.

**Table 3.** Generalized Difference-in-Differences Analysis of the Association Between Medicaid Expansion and Likelihood of Stage I or II Non–Small Cell Lung Cancer Diagnosis^a^

Term	OR (95% CI)	*P* value
Intercept	1.08 (1.03-1.14)	.004
Medicaid expansion status (expansion odds)	1.12 (1.05-1.18)	<.001
Time period (postpolicy odds)	0.91 (0.84-0.99)	.02
Expansion × postpolicy odds	1.07 (0.97-1.17)	.19

Abbreviation: OR, odds ratio.

^a^ The model includes Medicaid expansion status (expansion odds), representing baseline differences between expansion and nonexpansion states prior to policy implementation; time period (postpolicy odds), capturing secular temporal trends in early-stage diagnosis; and interaction term (expansion × postpolicy odds), representing the difference-in-differences estimate of interest. The interaction term indicates no statistically significant increase in the odds of early-stage diagnosis attributable to Medicaid expansion after accounting for baseline trends. The time effect reflects a modest overall decline in stage I to II diagnoses over time, while the group effect reflects higher baseline early-stage diagnosis in expansion states. These results suggest that Medicaid expansion was not associated with additional improvement in early-stage detection in this matched cohort.

## Discussion

Understanding the impact of Medicaid expansion on lung cancer outcomes is critical given the high mortality, the disproportionate burden of the disease on vulnerable populations, and the role structural barriers play in timely diagnosis and treatment. In this cohort study, we expand on prior research demonstrating improved survival following Medicaid expansion for various cancers, including lung cancer.^14,15,17,19^ These studies, however, are limited by short follow-up or lack inference methods to isolate policy effects. Using 16 years of data, Medicaid expansion under the ACA was associated with reduced mortality for stage I to IIIA NSCLC. States expanding on or before 2014 showed lower 2- and 4-year mortality, with the on-time expansion group seeing the largest decrease (9.5% reduction in 2-year mortality) (Table 2). Early expansion states consistently had the longest survival, likely reflecting existing access advantages in those states.

No significant 2-year mortality reduction was seen in the late expansion group; however, a significant decrease was seen in 4-year mortality. This lack of early significance is not unexpected, as no group demonstrated decreases during the first 3 years of Medicaid expansion. However, a decrease in 4-year mortality is notable, possibly due to states having well-established expansion schemas to borrow from or a greater baseline unmet need. It is also likely that extended follow-up periods allowed trends to achieve statistical significance. Such patterns align with the time required for systemic shifts to occur, underscoring the potential importance of policy duration and consistency in relation to population-level outcome.^36,37,38^ While time lags are well described in public health interventions,^39,40^ this phenomenon has not been previously described in the context of large-scale health policy analyses in cancer.

While patients in expansion states had modestly higher odds of early-stage (stage I and II) diagnosis, DID interactions were nonsignificant, indicating this advantage did not grow relative to nonexpansion states (Table 3). This suggests survival gains were not associated with earlier detection. Although our findings diverge from those of prior studies reporting increased early-stage presentation after expansion,^10,14,18^ they nonetheless suggest that Medicaid expansion may improve universal access to comprehensive cancer care, such as timely surgery or coordinated follow-up and/or surveillance. This contrast may partly stem from our excluding patients with advanced disease, leaving a narrower cohort among whom larger staging trends might be missed. However, our findings underscore the critical role of insurance in enabling comprehensive, timely treatment for curable NSCLC, an impact likely extending beyond early detection alone.

Beyond mortality, our analysis also investigated differential policy impacts across sociodemographic groups. While no significant mortality benefit was observed for sex, racial, or rurality groups, Medicaid expansion uniquely benefited currently or previously married patients. Conversely, benefits did not extend to the poorest counties (quintiles 1 and 2), with residents experiencing higher mortality hazards when referenced to the highest income quintile (quintile 5), while those in wealthier counties (quintiles 3 and 4) saw no change in mortality. This suggests that while Medicaid expansion may alleviate some financial barriers for enrolled individuals, it may not sufficiently address structural disadvantages faced by underserved populations and may perpetuate existing disparities.

Despite this, considering the overall positive impact, these findings suggest state-level policy reforms may decrease mortality. By expanding Medicaid eligibility, the ACA likely reduced financial and structural barriers to comprehensive care. Beyond individual benefits, Medicaid expansion significantly reduced uncompensated care costs and bolstered safety-net institution finances, indirectly enhancing capacity for complex cancer care and contributing to observed population-level mortality reductions.^41,42^ While modest mortality reductions confer substantial population-level impact, more predominant benefits observed in wealthier counties highlight the need for further work to alleviate socioeconomic disparities.^24,25,43^

### Strengths and Limitations

This study offers several strengths: natural policy experimentation, rigorous confounder adjustment, a placebo-falsification framework, and evaluation across 4clinical policy cohorts. Our time frame of longer than 1 decade and multiple expansion groups enhance generalizability and permit evaluation of policy timing and magnitude. Our DID models are robust to broader nationwide changes in practice (eg, immunotherapies, screening recommendations).^44,45^ Resectable NSCLC serves as a highly relevant model to examine Medicaid expansion’s impact, given its socioeconomic and demographic ties to diagnosis and survival.^20,21,22,23,24,25^

However, our findings should be interpreted within the context of several limitations. Retrospective cohort studies cannot infer causality. SEER’s lack of granular patient-level data, such as smoking, comorbidities, and insurance status, means unmeasured confounding may remain despite rigorous matching. Despite matching, unmeasured state differences may also serve as confounders. SEER’s limitation of insurance-level data further limits our analysis, as this cohort included patients with consistent private insurance coverage who were not directly impacted by the policy. Their inclusion likely biases our estimates toward the null, suggesting the true association between Medicaid expansion and improved survival may be stronger for Medicaid enrollees. Additionally, the inclusion of individuals who are younger than 65 years but receive Medicare due to disability or end-stage kidney disease, while a small proportion of the overall cohort, may introduce some bias. Furthermore, the inconsistent reporting of treatment variables in SEER precluded a direct assessment of whether improved survival was mediated by higher rates of guideline-concordant care.

Our cohort design itself presents important limitations. By focusing on resectable (stage I-IIIA) cancers, we could not assess whether expansion shifted diagnoses from stage IV to earlier stages, potentially underestimating total policy impact on presentation. While placebo-falsification analyses support stable pre-expansion patterns, this limits direct attribution of stage shifts to Medicaid expansion. Follow-up was limited to 4 years, possibly missing longer-term trends, and future work is needed to disentangle these complex policy effects over a longer duration.

We must also note the dynamic nature of health policy. While our findings highlight the mortality benefits of expansion, newly passed legislation may restrict Medicaid eligibility. Such policy reversals risk eroding the mortality decreases we observed and may disproportionately harm vulnerable populations with resectable NSCLC who stand to benefit from stable insurance coverage.

## Conclusions

In this cohort study, Medicaid expansion was associated with decreased 2- and 4-year mortality in patients with resectable NSCLC, with delayed but sustained decreases emerging over time. While causality cannot be inferred, these findings highlight the role of health policy in supporting access to complex cancer care. As debates over Medicaid continue, these findings suggest that long-term survival improvements may be achievable through early and comprehensive health policy implementation.

## Notes

Supplement 1. **eFigure 1.** US Map of State Medicaid Expansion Classification for States From the SEER Database **eTable 1.** SEER Database States Grouped by Time of Medicaid Expansion **eTable 2.** Baseline Characteristics of Patients With Resectable NSCLC by Medicaid Expansion Status **eFigure 2.** Survival Curves of Whole Unadjusted Groups Across Each Study Era **eTable 3.** Results of Pairwise Log-Rank Tests Evaluating Differences in Overall Survival Between Medicaid Expansion Groups **eFigure 3.** Plotted Distribution of Propensity Scores for Each Medicaid Expansion Group Against the Control Before (Above) and After (Below) Matching **eFigure 4.** Covariate Balance Before and After Propensity Score Matching **eFigure 5.** Survival Curves of Propensity-Matched Populations Across Each Study Era **eTable 4.** Results of Pairwise Log-Rank Tests Evaluating Differences in Overall Survival Between Medicaid Expansion Groups After Propensity Score Matching **eTable 5.** Placebo Falsification Model for Difference in Difference for Overall Survival **eFigure 6.** Placebo Difference-in-Differences Analysis to Assess for Spurious Trends in the Pre-Expansion Period **eFigure 7.** Trends in Average Survival by Medicaid Expansion Group (Matched Cohorts) **eTable 6.** Adjusted 2-, 4-, and 5-Year Mortality Hazards Across Medicaid Expansion Groups by Implementation Era **eTable 7.** Population Remaining Under Observation at Each Time Point in the Propensity Score Matched Cohort **eTable 8.** Multivariable Cox Regression Model for 2-Year Mortality in the Matched Cohort, Estimating Independent Associations of Demographic and Clinical Characteristics With Survival **eTable 9.** Subgroup Variation in Survival Benefit From Medicaid Expansion **eTable 10.** Placebo Falsification Model for Difference in Difference for Stage 1 and 2 Diagnosis **eTable 11.** Time-Treatment Interaction **eTable 12.** Royston-Parmar Parametric Model
eReferences

Supplement 2.
Data Sharing Statement
