# Supplementary material for: Statewide Medicaid Expansion and Survival in Resectable Non–Small Cell Lung Cancer
Source: JAMA Netw Open. 2025 Dec 1;8(12):e2545996. doi: 10.1001/jamanetworkopen.2025.45996 (PMC12670197; doi:10.1001/jamanetworkopen.2025.45996)
Supplement: Supplement 1. — eFigure 1. US Map of State Medicaid Expansion Classification for States From the SEER Database eTable 1. SEER Database States Grouped by Time of Medicaid Expansion eTable 2. Baseline Characteristics of Patients With Resectable NSCLC by Medicaid Expansion Status eFigure 2. Survival Curves of Whole Unadjusted Groups Across Each Study Era eTable 3. Results of Pairwise Log-Rank Tests Evaluating Differences in Overall Survival Between Medicaid Expansion Groups eFigure 3. Plotted Distribution of Propensity Scores for Each Medicaid Expansion Group Against the Control Before (Above) and After (Below) Matching eFigure 4. Covariate Balance Before and After Propensity Score Matching eFigure 5. Survival Curves of Propensity-Matched Populations Across Each Study Era eTable 4. Results of Pairwise Log-Rank Tests Evaluating Differences in Overall Survival Between Medicaid Expansion Groups After Propensity Score Matching eTable 5. Placebo Falsification Model for Difference in Difference for Overall Survival eFigure 6. Placebo Difference-in-Differences Analysis to Assess for Spurious Trends in the Pre-Expansion Period eFigure 7. Trends in Average Survival by Medicaid Expansion Group (Matched Cohorts) eTable 6. Adjusted 2-, 4-, and 5-Year Mortality Hazards Across Medicaid Expansion Groups by Implementation Era eTable 7. Population Remaining Under Observation at Each Time Point in the Propensity Score Matched Cohort eTable 8. Multivariable Cox Regression Model for 2-Year Mortality in the Matched Cohort, Estimating Independent Associations of Demographic and Clinical Characteristics With Survival eTable 9. Subgroup Variation in Survival Benefit From Medicaid Expansion eTable 10. Placebo Falsification Model for Difference in Difference for Stage 1 and 2 Diagnosis eTable 11. Time-Treatment Interaction eTable 12. Royston-Parmar Parametric Model eReferences [file jamanetwopen-e2545996-s001.pdf]

Supplementary Online Content

Gawdi R, Islam S, Sha C, et al. Statewide Medicaid expansion and survival in resectable non–small cell lung cancer. *JAMA Netw Open.* 2025;8(12):e2545996. doi:10.1001/jamanetworkopen.2025.45996

eFigure 1. US Map of State Medicaid Expansion Classification for States From the SEER Database
eTable 1. SEER Database States Grouped by Time of Medicaid Expansion
Table 2. Baseline Characteristics of Patients With Resectable NSCLC by Medicaid Expansion Status
eFigure 2. Survival Curves of Whole Unadjusted Groups Across Each Study Era
eTable 3. Results of Pairwise Log-Rank Tests Evaluating Differences in Overall Survival Between Medicaid Expansion Groups
eFigure 3. Plotted Distribution of Propensity Scores for Each Medicaid Expansion Group Against the Control Before (Above) and After (Below) Matching
eFigure 4. Covariate Balance Before and After Propensity Score Matching
eFigure 5. Survival Curves of Propensity-Matched Populations Across Each Study Era
eTable 4. Results of Pairwise Log-Rank Tests Evaluating Differences in Overall Survival Between Medicaid Expansion Groups After Propensity Score Matching
eTable 5. Placebo Falsification Model for Difference in Difference for Overall Survival
eFigure 6. Placebo Difference-in-Differences Analysis to Assess for Spurious Trends in the Pre-Expansion Period
eFigure 7. Trends in Average Survival by Medicaid Expansion Group (Matched Cohorts)
eTable 6. Adjusted 2-, 4-, and 5-Year Mortality Hazards Across Medicaid Expansion Groups by Implementation Era
eTable 7. Population Remaining Under Observation at Each Time Point in the Propensity Score Matched Cohort
eTable 8. Multivariable Cox Regression Model for 2-Year Mortality in the Matched Cohort, Estimating Independent Associations of Demographic and Clinical Characteristics With Survival
eTable 9. Subgroup Variation in Survival Benefit From Medicaid Expansion
eTable 10. Placebo Falsification Model for Difference in Difference for Stage 1 and 2 Diagnosis
eTable 11. Time-Treatment Interaction
eTable 12. Royston-Parmar Parametric Model
eReferences

This supplementary material has been provided by the authors to give readers additional information about their work.

© 2025 Gawdi R et al. *JAMA Network Open*.

Statewide Medicaid Expansion and Survival in Resectable Non–Small Cell Lung Cancer
Supplemental Materials

eFigure 1. US Map of State Medicaid Expansion Classification for States From the SEER Database

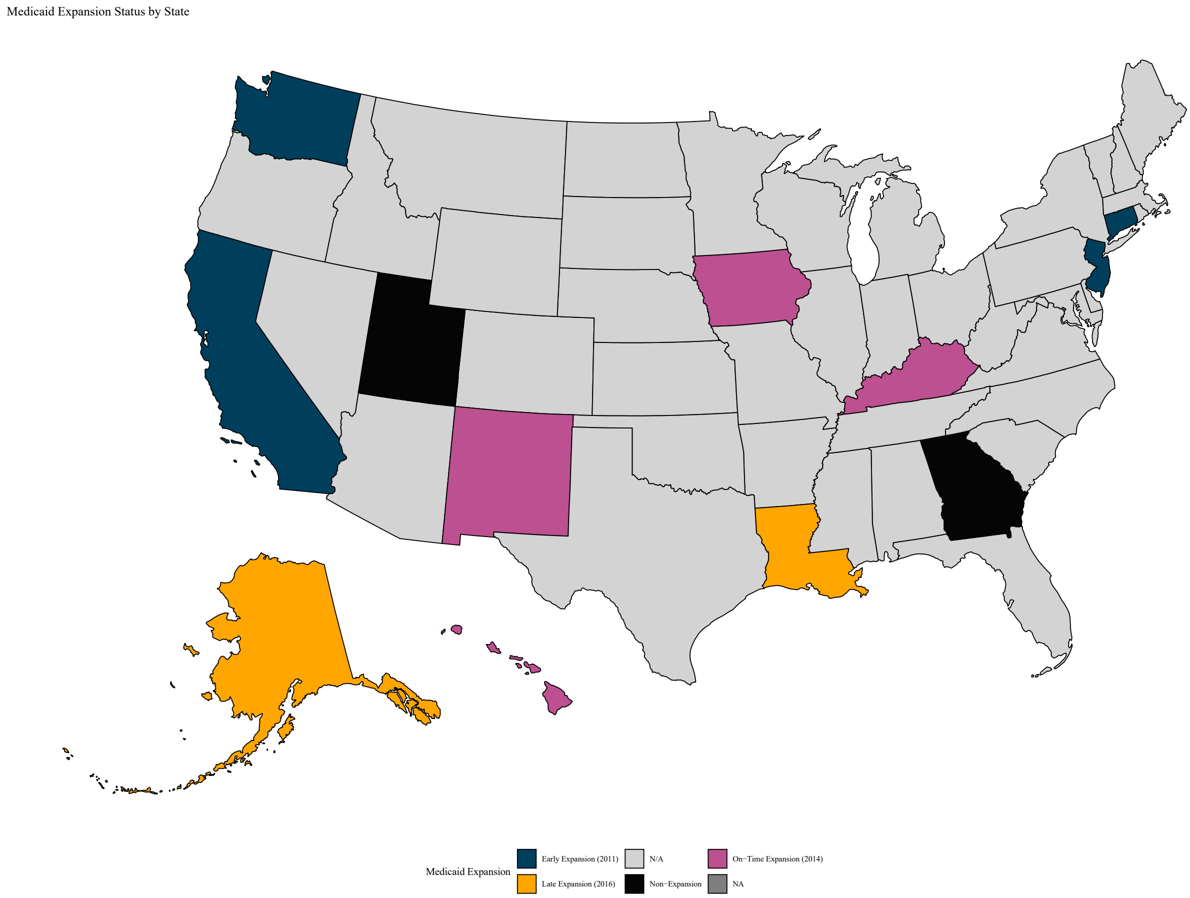

eFigure 1 presents a thematic map showing the classification of U.S. states by Medicaid expansion status for all states included in the SEER database. All states were grouped into either: Non-Expansion, Early Expansion (coverage expansion as of January 1, 2011 via Section 1115 waiver), 2014 Expansion (ACA implementation), and Late Expansion (post-2014). State categorization is based on the timing and mechanism of Medicaid policy change relative to the Affordable Care Act rollout and underpins the exposure variable in both unadjusted and adjusted analyses.

© 2025 Gawdi R et al. *JAMA Network Open*.

eTable 1. SEER Database States Grouped by Time of Medicaid Expansion

Expansion Status	Representative States in SEER Database
Non-Expansion	Utah*; Georgia
Early Expansion	California; Connecticut; New Jersey; Washington State** *(Used Section 1115 waivers to expand Medicaid per ACA as of 1/1/2011)*
2014 Expansion	Hawaii; Kentucky; Iowa; New Mexico
Late Expansion	Alaska (*expanded 9/1/2015)*; Louisiana *(expanded 7/1/2016)*

*Utah passed Medicaid expansion in 2020 and had accepted Section 1115 waivers to expand non-Medicaid insurance coverage prior to 2020.[1]
**Seattle (Puget Sound) is the representative region from Washington State in SEER Database.[2,3]

States and Medicaid enrollment in proceeding 3 years post-Expansion
California: 3.8 million enrollees (78.5% of newly eligible population)[4]
Connecticut: 46,000 enrollees[5]
New Jersey: 480,000 enrollees, reversing a trend of decreasing enrollment[6]
Washington State: 130,000 enrollees, however the 2009-2011 state budget cut funding to Washington's basic health plan by 43% and closed the program to new enrollees, thus Medicaid expansion was capped at 43,300/yr until full rollout of Medicaid Expansion was done in 2014.[5,7]
Hawaii: 215,000 enrollees[8]
Kentucky: 570,000 enrollees[9]
Iowa: 573,000 enrollees[10]
New Mexico: 460,000 enrollees[11]
Alaska: 75,000 enrollees [12]
Louisiana 350,000 enrollees[13]

Total – nearly 6.2 million patients successfully enrolled in Medicaid in the three years after each state expanded Medicaid.[1]

© 2025 Gawdi R et al. *JAMA Network Open*.

eTable 2.		No Expansion	Early Expansion	2014 Expansion	Late Expansion	p-value
Age	< 54yo	2383 (24.08%)	6835 (23.71%)	2609 (26.05%)	1219 (26.05%)	<0.001
	55-59yo	2990 (30.21%)	8387 (29.10 %)	3135 (30.02%)	1435 (30.67%)	
	60-64yo	4523 (45.71%)	13603 (47.19%)	4698 (44.99 %)	2025 (43.28%)	
Sex	Male	5157 (52.11%)	13800 (47.88%)	5550 (53.15%)	2520 (53.86%)	< 0.001
	Female	4739 (47.89%)	15025 (52.12%)	4892 (46.85%)	2159 (46.14%)	
Marital Status	Single/Unknown	2261 (22.85%)	7736 (26.84%)	1786 (17.10%)	1347 (28.79%)	<0.001
	Married	5086 (51.39%)	15144 (52.54%)	5578 (53.42%)	2239 (47.85%)	
	Previously Married	2549 (25.76%)	5945 (20.62%)	3078 (29.48%)	1093 (23.36%)	
Surgery	Recommended + performed	5588 (56.47%)	18948 (65.73%)	6312 (60.45%)	2311 (49.39%)	<0.001
	Not recommended + unknown	4053 (40.96%)	9263 (32.14%)	3942 (37.75%)	2255 (48.19%)	
	Recommended, not performed	255 (2.58%)	614 (2.13%)	188 (1.80%)	113 (2.42%)	
Radiotherapy	Received	3734 (37.73%)	8741 (30.32%)	4062 (38.90%)	2031 (43.41%)	<0.001
	None/unknown/refused	6162 (62.27%)	20084 (69.68%)	6380 (61.10%)	2648 (56.59%)	
Stage at Dx	I	5042 (50.95%)	15659 (54.32%)	5362 (51.35%)	2228 (47.62%)	<0.001
	II+III	4854 (49.05%)	13166 (45.68%)	5080 (48.65%)	2451 (52.38%)	
Rurality	Metropolitan > 1 million pop	4008 (40.50%)	20626 (71.56%)	1969 (18.86%)	1148 (24.54%)	<0.001
	Metropolitan 250k – 1m pop	1690 (17.08%)	5715 (19.83%)	2450 (23.46%)	1637 (34.99%)	
	Metropolitan <250k	1733 (17.51%)	1303 (4.52%)	1242 (11.89%)	947 (20.24%)	
	Nonmetropolitan Metro-adjacent	1690 (17.08%)	928 (3.22%)	1868 (17.89%)	814 (17.40%)	
	Nonmetropolitan Nonmetro-adjacent	775 (7.83%)	253 (0.88%)	2913 (27.90%)	133 (2.84%)	
Race	Non-Hispanic White	7179 (72.54%)	20340 (70.56%)	9174 (87.86%)	3030 (64.76%)	<0.001
	Hispanic (All Races)	378 (3.81%)	2459 (8.53%)	99 (0.95%)	32 (0.68%)	
	Non-Hispanic American Indian/Alaska Native	35 (0.35%)	203 (0.70%)	8 (0.08%)	12 (0.26%)	

© 2025 Gawdi R et al. *JAMA Network Open*.

	Non-Hispanic Asian or Pacific Islander	123 (1.24%)	2607 (9.04%)	560 (5.36%)	44 (0.94%)	
	Non-Hispanic Black	2178 (22.00%)	3124 (10.84%)	595 (5.70%)	1560 (33.34%)	
	Non-Hispanic Unknown Race	3 (0.03%)	92 (0.32%)	6 (0.06%)	1 (0.02%)	
Median County Income Range	< $35,000	4518 (45.65%)	1327 (4.60%)	5142 (49.24%)	2815 (60.16%)	<0.001
	$35,000 - $57,500	1981 (20.02%)	3148 (10.92%)	3025 (28.97%)	1306 (27.91%)	
	$57,500 - $77,500	1854 (18.73%)	8255 (28.64%)	1314 (12.58%)	398 (8.51%)	
	$57,500 - $92,500	1315 (13.29%)	7429 (25.77%)	695 (6.66%)	160 (3.42%)	
	> $92,500	228 (2.30%)	8666 (30.06%)	266 (2.55%)	0 (0)	

Table 2. Baseline Characteristics of Patients With Resectable NSCLC by Medicaid Expansion Status

eTable 2 presents baseline demographic, clinical, and socioeconomic characteristics of patients diagnosed with resectable non–small cell lung cancer (NSCLC), stratified by state Medicaid expansion status: non-expansion, Early Expansion, 2014 Expansion, and Late Expansion. Variables include age, sex, marital status, treatment modalities (surgery and radiotherapy), stage at diagnosis, urban-rural classification, race/ethnicity, and median county income quintile. All variables are assessed across the entire duration of the study (2006-2019). Significant variation is observed across groups, particularly in income distribution, rurality, racial composition, and treatment patterns, highlighting the importance of propensity score matching in adjusting for these imbalances. p-values are derived from chi-squared tests and indicate overall group differences for each covariate.

To generate the demographic table, several variables in the SEER dataset were grouped to facilitate comparison across Medicaid expansion groups. Year of diagnosis was grouped into four periods—2006–2010, 2011–2013, 2014–2016, and 2017–2019—based on national Medicaid expansion milestones. States were then classified into four Medicaid expansion categories (0–3) according to the timing of their expansion relative to each era: 0 for non-expanders (e.g. Georgia, Utah), 1 for early expanders (e.g., California, Connecticut, New Jersey, Washington State), 2 for on-time expanders (e.g., Iowa, Kentucky, New Mexico, Hawaii), and 3 for late expanders (e.g., Louisiana, Alaska). Age was treated as a categorical variable grouped in 5-year intervals. Cancer stage at diagnosis was collapsed into two broad categories: Stage I, Stage II+IIIA. Race/ethnicity, marital status at diagnosis, and rural/urban status were used as recorded in SEER but treated as categorical factors.

Household income was mapped from inflation-adjusted categorical brackets to continuous midpoints and then divided into population-level quintiles (Q1–Q5) to assess socioeconomic gradients across the entire nation. Income quintiles varied substantially between states due to differences in cost-of-living and were similarly not retained in final models. Radiation treatment was grouped into two categories: “Radiation received” and “None/unknown/refused,” combining beam radiation and brachytherapy-based modalities into the former. Similarly, surgical treatment was categorized into “Recommended + performed,” “Recommended, not performed,” and “Not recommended/unknown,” based on SEER’s surgery recode. Surgery and radiation receipt were included as clinical variables but were not retained in final models to avoid collinearity. Lastly, the outcome variables included a binary vital status indicator (alive vs deceased) and a continuous measure of overall survival time, which was capped at 24 months and cleaned as necessary for survival analysis.

© 2025 Gawdi R et al. *JAMA Network Open*.

eFigure 2. Survival Curves of Whole Unadjusted Groups Across Each Study Era

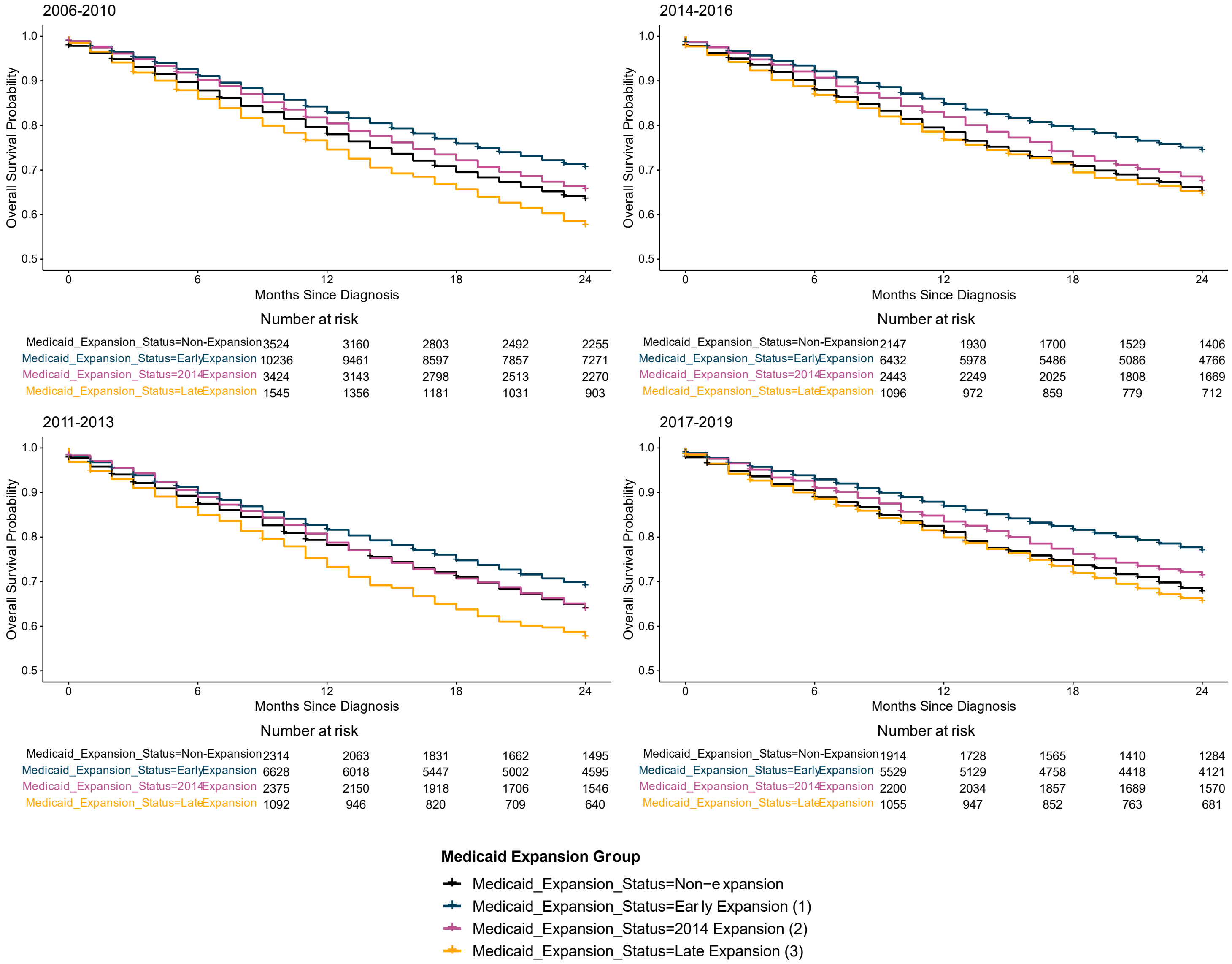

Survival Curves of Whole Unadjusted Groups Across Each Study Era

eFigure 2 shows Kaplan-Meier estimates of 2-year overall survival for patients with stage I–IIIA non–small cell lung cancer (NSCLC) across Medicaid expansion groups without adjustement or propensity score matching. Curves represent matched comparisons between non-expansion states and each of the following: Early Expansion (Group 1; via federal waivers as of 2011), 2014 Expansion (Group 2; ACA implementation), and Late Expansion (Group 3; post-2014). Survival time was capped at 24 months with event censoring.

© 2025 Gawdi R et al. *JAMA Network Open*.

eTable 3. Results of Pairwise Log-Rank Tests Evaluating Differences in Overall Survival Between Medicaid Expansion Groups

	2 Year Survival Rates (%)			
	2006-2010	2011-2013	2014-2016	2017-2019
Non-expansion	63.45	63.88	65.22	67.68
Early Expansion	70.49	68.99	74.29	76.81
2014 Expansion	65.61	63.80	67.44	71.31
Late Expansion	57.54	57.54	64.57	65.55
**p-value**	<0.001**	<0.001**	<0.001**	<0.001**

Pair-Wise Comparisons for Entire Cohort (Pre-Matching

2006-2010	Non-Exp	Early Exp	2014 Exp	Late Exp
Non-Exp		<0.001**	0.025	<0.001**
Early Exp	<0.001**		<0.001**	<0.001**
2014 Exp	0.025	<0.001**		<0.001**
Late Exp	<0.001**	<0.001**	<0.001**	

2011-2013	Non-Exp	Early Exp	2014 Exp	Late Exp
Non-Exp		<0.001**	0.89	<0.001
Early Exp	<0.001**		<0.001**	<0.001**
2014 Exp	0.89	<0.001**		<0.001**
Late Exp	<0.001	<0.001**	<0.001**	

2014-2016	Non-Exp	Early Exp	2014 Exp	Late Exp
Non-Exp		<0.001**	0.13	0.62
Early Exp	<0.001**		<0.001**	<0.001**
2014 Exp	0.13	<0.001**		0.13
Late Exp	0.62	<0.001**	0.13	

2017-2019	Non-Exp	Early Exp	2014 Exp	Late Exp
Non-Exp		<0.001**	0.02	0.26
Early Exp	<0.001**		<0.001**	<0.001**
2014 Exp	0.02	<0.001**		0.002
Late Exp	0.26	<0.001**	0.002	

All values Benjamini-Hochberg adjusted

eTable 3 presents the results of pairwise log-rank tests evaluating differences in overall survival between Medicaid expansion groups (non-expansion, early expansion, 2014 expansion, and late expansion) within each study era. Separate analyses were conducted for each era to assess whether survival distributions differed significantly between groups over time. To account for multiple comparisons and reduce the false discovery rate, p-values were adjusted using the Benjamini-Hochberg procedure. Adjusted q-values are presented, with * indicating statistical significance at $q < 0.05$ and ** at $q < 0.001$. These comparisons provide complementary evidence to the survival curves and Cox models, supporting nuanced interpretation of survival differences between expansion groups across eras.

© 2025 Gawdi R et al. *JAMA Network Open*.

eFigure 3. Plotted Distribution of Propensity Scores for Each Medicaid Expansion Group Against the Control Before (Above) and After (Below) Matching

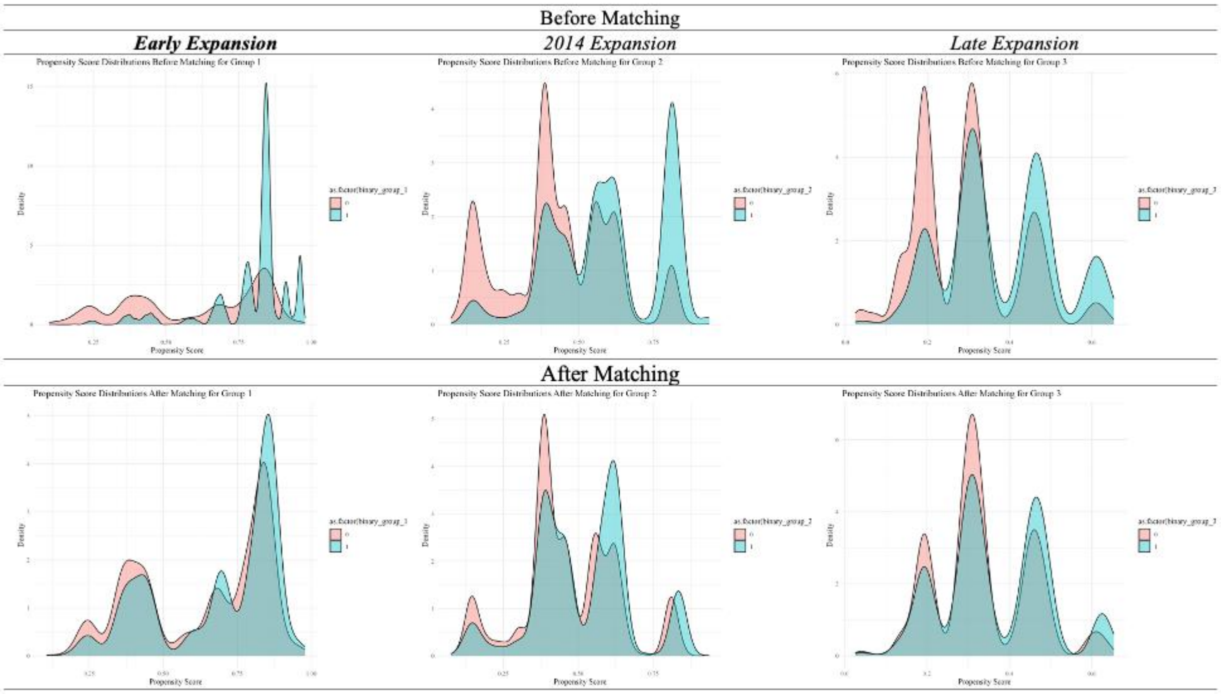

eFigure 3 shows the plotted distribution of propensity scores for each Medicaid expansion group against the control before (above) and after (below) matching. We apply 1:1 nearest-neighbor matching without replacement and with caliper width of 0.2 standard deviations to optimize match quality and sample retention. Matching was performed within each expansion group (blue) against non-expansion controls (pink). Overlap between distributions improves substantially post-matching, demonstrating successful balancing of observed covariates used in the propensity score model.

To optimize match quality and minimize bias, we used the logit of the propensity score (rather than the raw propensity score) when applying caliper-based nearest-neighbor matching. This transformation stretches values near 0 and 1, making the distance metric more sensitive and reducing the likelihood of poor matches, particularly for individuals with extreme scores.

A caliper width of 0.2 standard deviations on the logit scale was used, which is a commonly recommended threshold to balance precision and sample retention. Matching was performed without replacement within each expansion group and era, and matching quality was assessed using standardized mean differences.

This approach improved covariate balance across key demographic and clinical variables, enhancing the validity of comparisons between treated and control groups. The use of the logit scale is a standard best practice when implementing caliper matching with propensity scores.

© 2025 Gawdi R et al. *JAMA Network Open*.

eFigure 4. Covariate Balance Before and After Propensity Score Matching

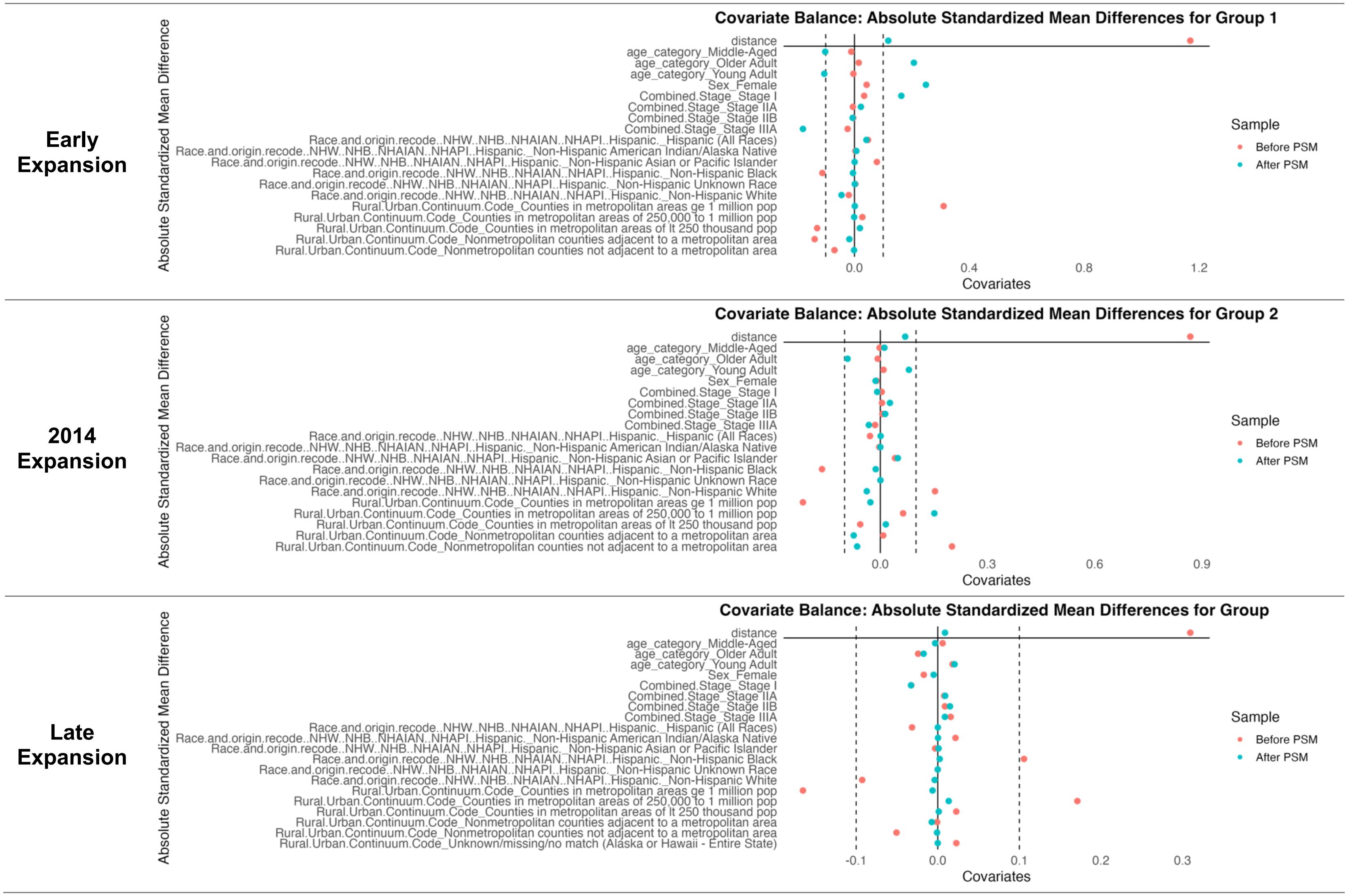

© 2025 Gawdi R et al. *JAMA Network Open*.

.

eFigure 4 presents standardized mean differences for key covariates in each expansion group before (red dots) and after (blue dots) 1:1 propensity score matching without replacement. Each panel corresponds to a specific Medicaid expansion group and era comparison. Post-matching covariate balance improved substantially, with most standardized differences falling below the conventional threshold of 0.1, indicating adequate adjustment for observed confounding. Notably, achieving adequate balance for the Early Expansion vs Non-Expansion comparison was more challenging due to limited overlap in demographic and socioeconomic profiles, resulting in mean covariate balance distance slightly beyond optimal (0.1178). Further attempted restrictive matching parameters result in overfitting, obscuring meaningful group differences.

© 2025 Gawdi R et al. *JAMA Network Open*.

eFigure 5. Survival Curves of Propensity-Matched Populations Across Each Study Era

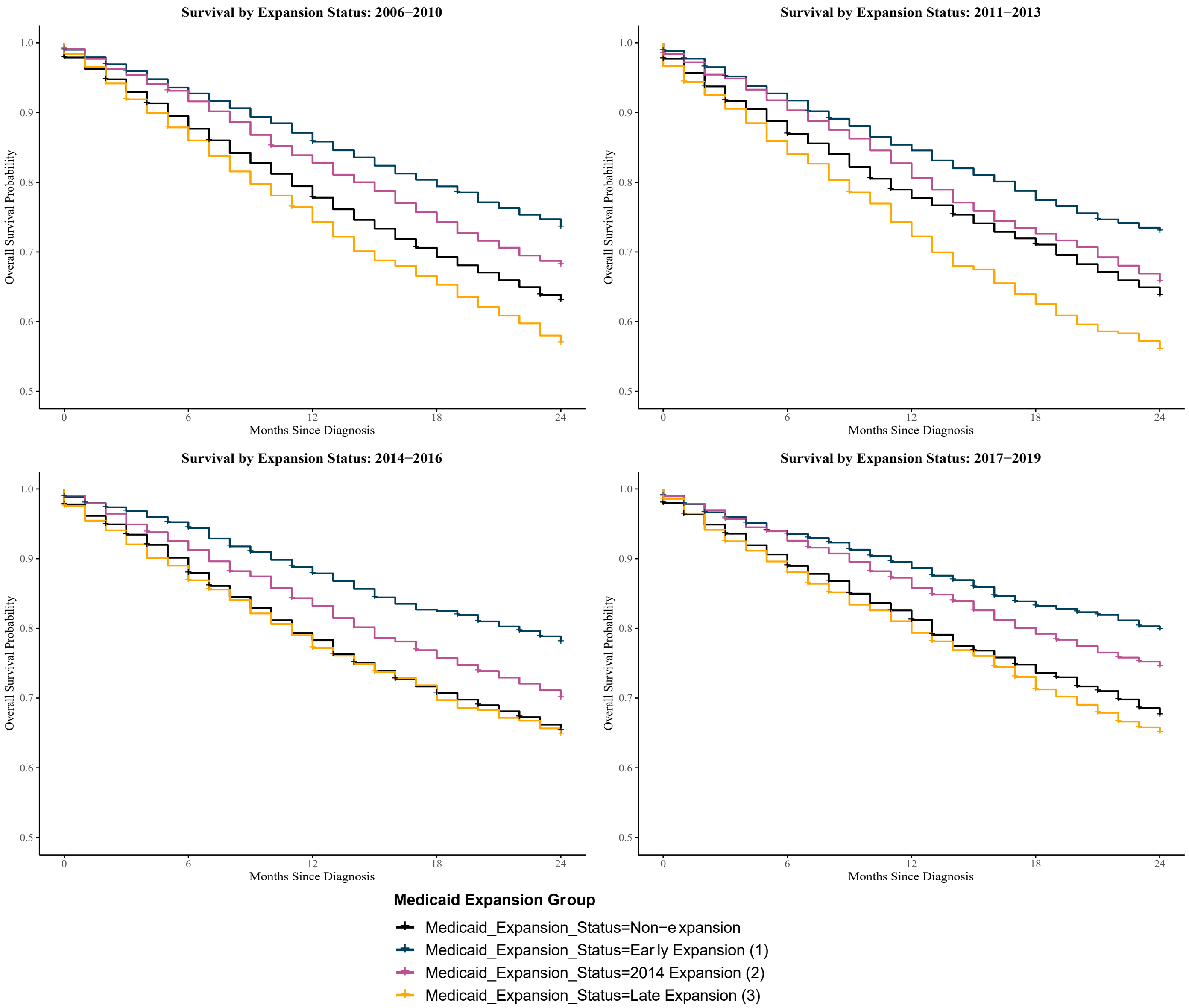

eFigure 5 shows Kaplan-Meier estimates of 2-year overall survival for patients with stage I–IIIA non–small cell lung cancer (NSCLC) across Medicaid expansion groups after propensity score matching. Curves represent matched comparisons between non-expansion states and each of the following: Early Expansion (Group 1; via federal waivers as of 2011), 2014 Expansion (Group 2; ACA implementation), and Late Expansion (Group 3; post-2014). Survival time was capped at 24 months, and matching was performed to balance key demographic, socioeconomic, and clinical covariates.

© 2025 Gawdi R et al. *JAMA Network Open*.

eTable 4. Results of Pairwise Log-Rank Tests Evaluating Differences in Overall Survival Between Medicaid Expansion Groups After Propensity Score Matching

	2 Year Survival Rates (%)			
	**2006-2010**	**2011-2013**	**2014-2016**	**2017-2019**
Non-expansion	65.00	65.98	67.00	68.52
Early Expansion	75.28	74.03	78.75	78.53
2014 Expansion	69.28	67.97	71.54	75.09
Late Expansion	58.95	59.58	66.84	65.35
p-values	<0.001**	<0.001**	<0.001**	<0.001**

Pair-Wise Comparisons for of Survival for Propensity Score Matched Populations									
2006-2010					2011-2013				
	Non-Exp	Early Exp	2014 Exp	Late Exp		Non-Exp	Early Exp	2014 Exp	Late Exp
Non-Exp		<0.001**		<0.001**	Non-Exp		<0.001**	0.04	<0.001**
Early Exp	<0.001**		<0.001**	<0.001**	Early Exp	<0.001**		<0.001**	<0.001**
2014 Exp	<0.001**	<0.001**		<0.001**	2014 Exp	0.04	<0.001**		<0.001**
Late Exp	<0.001**	<0.001**	<0.001**		Late Exp	<0.001**	<0.001**	<0.001**	

2014-2016					2017-2019				
	Non-Exp	Early Exp	2014 Exp	Late Exp		Non-Exp	Early Exp	2014 Exp	Late Exp
Non-Exp		<0.001**	<0.001	0.684	Non-Exp		<0.001**	<0.001**	0.14
Early Exp	<0.001**		<0.001**	<0.001**	Early Exp	<0.001**		0.001	<0.001**
2014 Exp	<0.001	<0.001**		0.0024	2014 Exp	<0.001**	0.001		<0.001**
Late Exp	0.684	<0.001**	0.0024		Late Exp	0.14	<0.001**	<0.001**	

eTable 4 presents the results of pairwise log-rank tests evaluating differences in overall survival between Medicaid expansion groups (non-expansion, early expansion, 2014 expansion, and late expansion) after propensity score matching across each study era. Separate analyses were conducted for each era to assess whether survival distributions differed significantly between groups over time. To account for multiple comparisons and reduce the false discovery rate, p-values were adjusted using the Benjamini-Hochberg procedure. Adjusted q-values are presented, with * indicating statistical significance at $q < 0.05$ and ** at $q < 0.001$. These comparisons provide complementary evidence to the post-matching survival curves and Cox models, supporting nuanced interpretation of survival differences between expansion groups across eras.

© 2025 Gawdi R et al. *JAMA Network Open*.

eTable 5. Placebo Falsification Model for Difference in Difference for Overall Survival

Variable	Coefficient Estimate	Standard Error	t-value	p-value
**Intercept**	19.16	0.11	178.275	<0.001
**DiD Treat**	1.07	0.15	7.02	<0.001
**Placebo Time**	-0.22	0.24	-0.91	0.40
**DiD Treat x Placebo Time**	–0.06	0.34	–0.19	0.86

Model Fit:
Residual SE: 0.21 on 6 DF
Multiple R^2: 0.91
Adjusted R^2: 0.87
F-statistic: 20.78 on 3 and 6 DF, p-value: 0.001

eTable 5 shows results of a placebo falsification model to assess parallel trends in 2-year survival for NSCLC. Placebo time was defined by artificially treating 2010 as a "post-expansion" timepoint. We then conducted a difference-in-differences analysis restricted to the pre-expansion period (2006–2010). The model was fit using group-level averages (i.e., mean 2-year capped survival by state group and year), comparing early expansion versus non-expansion states. The placebo model explained a high proportion of variance (R^2 = 0.91), and overall model fit was strong (F(3,6) = 20.78, P = 0.001). While early expansion states had significantly higher average survival at baseline (β = 1.07, P = 0.0004), the interaction term—representing the placebo difference-in-differences effect—was small and not statistically significant (β = –0.06, P = 0.86). These results indicate that trends in survival between the two groups were parallel prior to expansion, providing reassurance that our main findings are unlikely to be driven by underlying baseline differences or differential pre-expansion trends.

The model showed excellent overall fit, with an R^2 of 0.912, indicating that 91% of the variance in group-level capped survival was explained by the model. The adjusted R^2 remained high at 0.868, accounting for the small number of groups (n = 10). The F-statistic was 20.78 (3, 6 degrees of freedom) with a P-value of 0.0014, suggesting the overall model was statistically significant.

Importantly, the placebo interaction term (treat × placebo time) was small and non-significant (P = 0.86), consistent with the absence of differential trends in survival prior to Medicaid expansion. These findings support the validity of the parallel trends assumption for our main DiD analysis.

eFigure 6. Placebo Difference-in-Differences Analysis to Assess for Spurious Trends in the Pre-Expansion Period

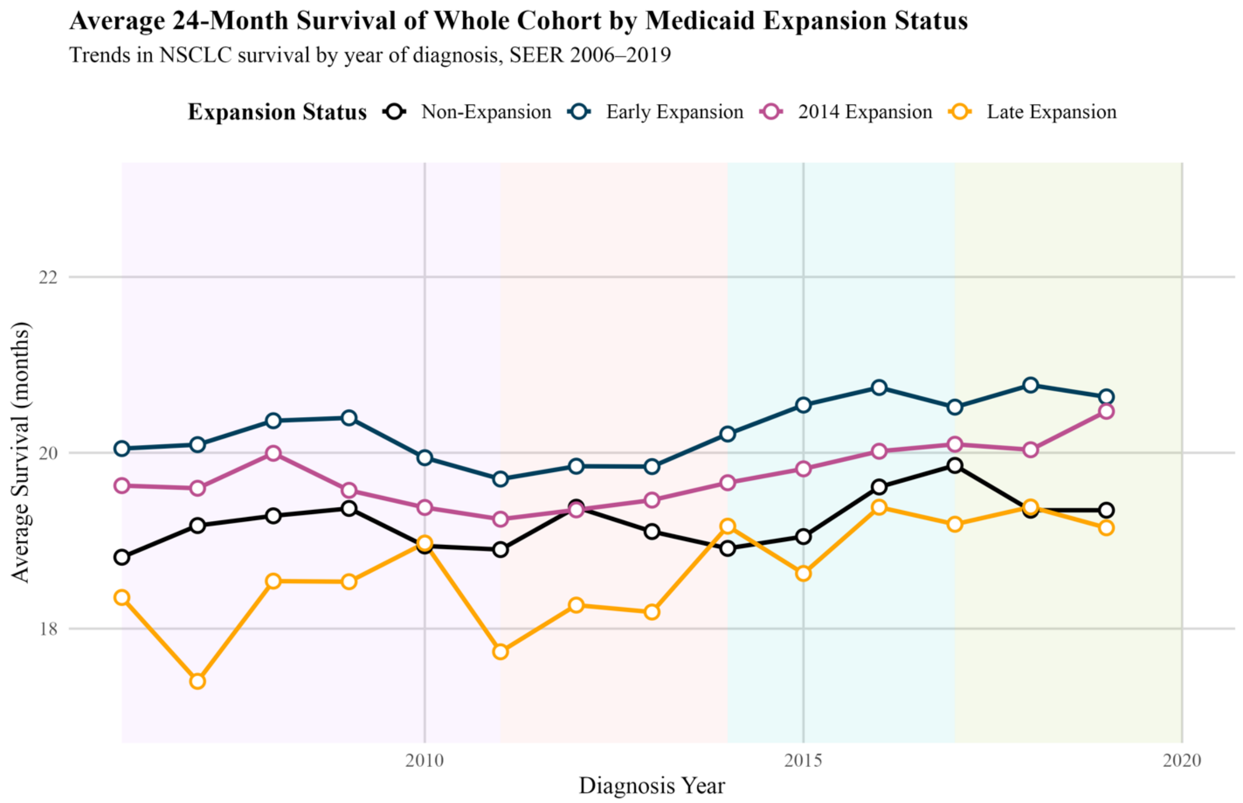

eFigure 6 displays graphical results from a falsification test using a placebo difference-in-differences (DiD) model. Annual estimates of 2-year overall survival among patients with resectable non–small cell lung cancer (NSCLC) are plotted across Medicaid expansion and non-expansion states over the entire study duration. This test evaluates the parallel trends assumption by confirming that survival trends between groups were not diverging before Medicaid expansion. The absence of significant differences in survival slopes between groups before each policy transition year (2011, 2014, 2016) supports the validity of the DiD approach used in the primary analysis.

© 2025 Gawdi R et al. *JAMA Network Open*.

eFigure 7. Trends in Average Survival by Medicaid Expansion Group (Matched Cohorts)

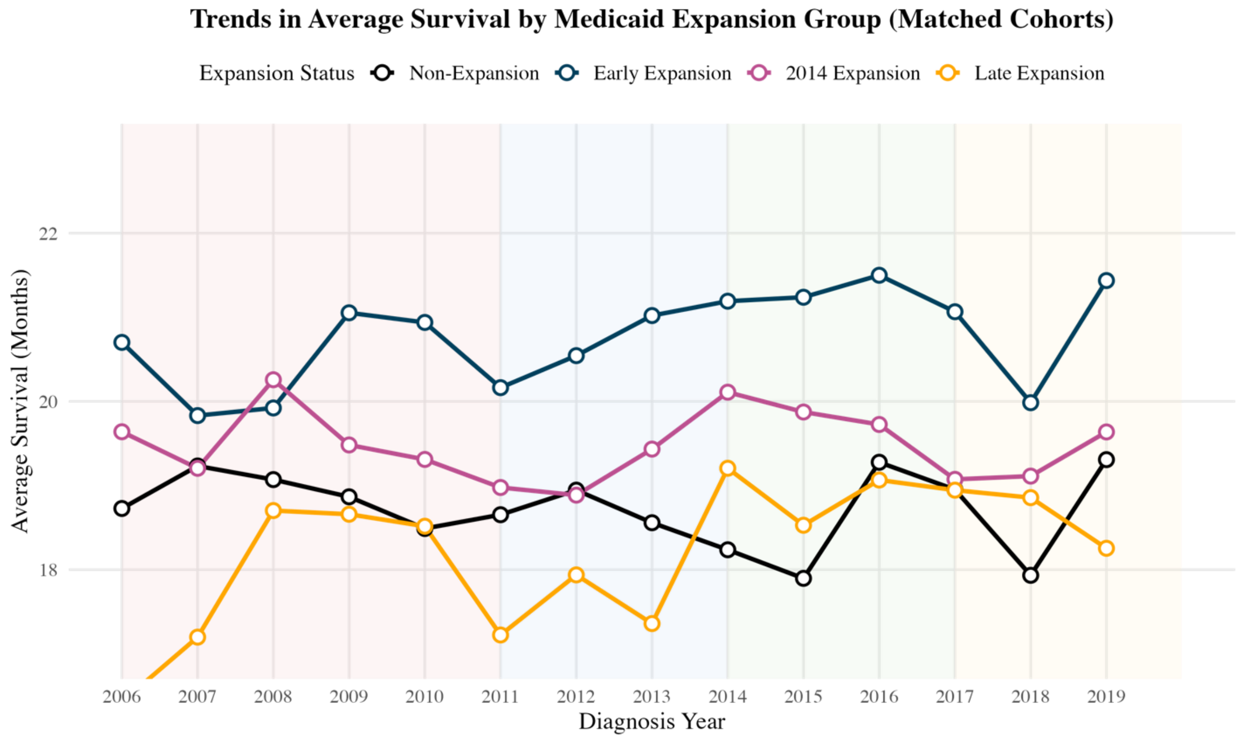

Average Capped Survival by Diagnosis Year and Medicaid Expansion Status (Propensity Score–Matched Cohorts).

eFigure 7 displays graphical results showing annual estimates of 2-year overall survival among patients in our resectable NSCLC cohort after propensity score matching. Survival time (capped at 24 months) is plotted across Medicaid expansion and non-expansion states over the entire study duration. This test further validates the results of our matching algorithm, and visually demonstrates evidence of parallel trends in survival for the propensity-score matched groups. The absence of significant differences in survival slopes between groups before each policy transition year (2011, 2014, 2016) supports the validity of the DiD approach used in the primary analysis.

© 2025 Gawdi R et al. *JAMA Network Open*.

eTable 6. Adjusted 2-, 4-, and 5-Year Mortality Hazards Across Medicaid Expansion Groups by Implementation Era

	**Era**	**HR (95% CI)**	**p-value**	**HR (95% CI)**	**p-value**	**HR (95% CI)**	**p-value**
		2-Year Survival		4-Year Survival		5-Year Survival	
**Early Expansion**	Pre-Expansion (2006-2010)	---	---	---	---		---
	Implementation (2011-2013)	1.07 (0.97–1.17)	0.17	1.07 (0.99-1.14)	0.06	1.07 (0.99-1.16)	0.09
	Post-Implementation (2014-2019)	0.89 (0.85–0.93)	*<0.001*	*0.91 (0.89-0.94)*	*<0.001*	*0.94 (0.90-0.99)*	*0.02*
	Total Post-Expansion Period	0.95 (0.91–0.99)	*0.020*	*0.97 (0.94-0.99)*	*0.03*	0.98 (0.95-1.03)	0.60
**2014 Expansion**	Pre-Expansion (2006-2013)	---	---	---	---		---
	Implementation (2014-2016)	0.95 (0.88–1.03)	0.25	*0.97 (0.95-0.99)*	*0.004*	0.99 (0.96-1.02)	0.494
	Post-Implementation (2017-2019)	0.85 (0.77–0.94)	*0.001*	*0.86 (0.80-0.93)*	*<0.001*	*0.86 (0.79-0.93)*	*<0.001*
	Total Post-Expansion Period	0.91 (0.86–0.95)	*<0.001*	*0.92 (0.89-0.95)*	*<0.001*	*0.93 (0.90-0.96)*	*<0.001*
**Late Expansion**	Pre-Expansion (2006-2016)	---	---	---	---	---	---
	Implementation (2017-2019)	0.95 (0.89–1.02)	0.15	*0.92 (0.89-0.94)*	*<0.001*	*0.93 (0.90-0.95)*	*<0.001*

eTable 6 shows the adjusted hazard ratios for mortality censored at 2, 4, and 5 years across Medicaid expansion groups and time periods, using generalized Difference in difference models using the propensity score matched cohort. Each panel represents a distinct expansion group, with eras defined as pre-expansion, implementation, and post-implementation.

Five-year mortality HRs are provided in this supplement to contextualize long-term effects but were excluded from the main text due to underrepresentation of diagnoses in the post-implementation eras, underweighting patients most likely to show benefit. Thus, inclusion of 5-year outcomes in the primary figures risked misrepresenting data maturity and censoring bias. Thus, the main manuscript focuses on 2- and 4-year survival, which provide more reliable and generalizable estimates across all groups.

© 2025 Gawdi R et al. *JAMA Network Open*.

eTable 7. Population Remaining Under Observation at Each Time Point in the Propensity Score Matched Cohort

Expansion Status	N (Total)	24 months	% Remaining	48 months	% Remaining	60 months	% Remaining
**Non-Expansion**	25160	16344	65.0%	10828	43.0%	8908	35.4%
**Early Expansion**	8817	6128	75.5%	4310	53.1%	3641	44.9%
**2014 Expansion**	6837	4779	69.9%	3160	46.2%	2520	36.9%
**Late Expansion**	4426	2688	60.7%	1662	37.6%	1346	30.4%

eTable 7 shows the number and percentage of patients remaining under observation (i.e., not censored) at 24, 48, and 60 months following diagnosis in the propensity score matched population. These figures provide context for mortality estimates at each time point and highlight differences in follow-up duration across Medicaid expansion groups. Lower proportions of uncensored individuals at later time points—particularly in the late expansion and non-expansion cohorts—reflect expected administrative censoring and reduced data maturity for recent diagnoses.

© 2025 Gawdi R et al. *JAMA Network Open*.

eTable 8. Multivariable Cox Regression Model for 2-Year Mortality in the Matched Cohort, Estimating Independent Associations of Demographic and Clinical Characteristics With Survival

		HR	CI_lower	CI_upper	p-value
**Age**	60-64 years old	0.99	0.92	1.06	0.66
	54 years old	0.87	0.79	0.95	0.001
**Sex**	Female	0.69	0.65	0.71	<0.001
**Stage**	II + IIIa	2.34	2.21	2.47	<0.001
**Marital Status**	Married + Domestic Partner	0.76	0.71	0.80	<0.001
	Previously Married	0.98	0.90	1.07	0.64
**Rurality**	Metropolitan 250k – 1m pop	1.12	1.01	1.25	0.03
	Metropolitan <250k	1.21	1.04	1.41	0.01
	Nonmetropolitan Metro-adjacent	1.31	1.13	1.52	<0.001
	Nonmetropolitan Nonmetro-adjacent	1.23	1.08	1.39	<0.001
	Unknown	1.57	0.70	3.51	0.28
**Race**	Hispanic (All Races)	1.09	0.94	1.27	0.26
	Non-Hispanic American Indian/Alaska Native	0.72	0.31	1.67	0.44
	Non-Hispanic Asian or Pacific Islander	0.49	0.32	0.77	0.002
	Non-Hispanic Black	1.00	0.88	1.13	0.96

eTable 8 presents the covariates independently associated with 2-year overall survival from the Cox proportional hazards model fit to the combined propensity score–matched cohorts. The analysis includes demographic, socioeconomic, and geographic factors across all matched groups. Reference categories for categorical variables are as follows:

- *Age:* 55–59 years
- *Marital Status:* Single, never married
- *Urban-Rural Classification:* Metropolitan area with population >1 million
- *Race/Ethnicity:* Non-Hispanic White
- *Stage:* Stage I

Hazard ratios (HR) less than 1 indicate improved decreased mortality hazard compared with the reference groups. The model accounts for Medicaid expansion group clustering and includes robust standard errors to address within-group correlation

© 2025 Gawdi R et al. *JAMA Network Open*.

eTable 9. Subgroup Variation in Survival Benefit From Medicaid Expansion

	term	HR	Lower CI	Upper CI	p-value	sig
**Age**	55-59	0.95	0.87	1.03	0.18	No
	<54 years old	1.02	0.94	1.12	0.60	No
**Sex**	Male	0.98	0.92	1.06	0.67	No
**Marital Status**	Married + Domestic Partner	0.91	0.84	0.99	0.04	Yes
	Previously Married	0.90	0.81	0.99	0.03	Yes
**Race**	Hispanic (All Races)	0.94	0.80	1.11	0.48	No
	Non-Hispanic American Indian/Alaska Native	1.21	0.71	2.05	0.49	No
	Non-Hispanic Asian or Pacific Islander	0.84	0.706	1.00	0.05	Yes
	Non-Hispanic Black	0.92	0.826	1.03	0.13	No
**Rurality**	Metropolitan 250k – 1m pop	1.00	0.91	1.09	0.93	No
	Metropolitan <250k	0.91	0.79	1.05	0.20	No
	Nonmetropolitan Metro-adjacent	0.901	0.78	1.05	0.18	No
	Nonmetropolitan Nonmetro-adjacent	0.90	0.77	1.06	0.20	No
**County Income Quintile**	Quintile 1	1.19	1.03	1.38	0.02	Yes
	Quintile 2	1.16	1.03	1.32	0.02	Yes
	Quintile 3	1.03	0.92	1.15	0.62	No
	Quintile 4	1.03	0.92	1.16	0.64	No

eTable 9 presents adjusted hazard ratios for mortality within the propensity score–matched cohort, stratified by demographic and geographic characteristics to assess heterogeneity in response to Medicaid expansion. Expansion was associated with greater survival among patients who were married or previously married and those residing in higher-income counties. No significant differential benefit was observed by sex, race/ethnicity, or rurality.

© 2025 Gawdi R et al. *JAMA Network Open*.

eTable 10. Placebo Falsification Model for Difference in Difference for Stage 1 and 2 Diagnosis

Variable	Coefficient Estimate	Standard Error	t-value	p-value
**Intercept**	0.55	0.02	31.46	<0.001
**DiD Treat**	0.03	0.03	1.29	0.25
**Placebo Time**	-0.07	0.04	-1.83	0.12
**DiD Treat x Placebo Time**	–0.005	0.06	–0.08	0.94

Model Fit:
Residual SE: 0.03 on 6 DF
Multiple R-squared: 0.60
Adjusted R-squared: 0.40
F-statistic: 3.03 on 3 and 6 DF,
p-value: 0.12

eTable 10 shows results of a placebo falsification model examined trends in the proportion of early-stage diagnoses prior to Medicaid expansion (2006–2010), grouped by expansion status and year. The model demonstrated moderate explanatory power, with an R^2 of 0.60, indicating that approximately 60.3% of the variance in the group-level early-stage diagnosis rate was accounted for by the model. The adjusted R^2 was lower (0.40), reflecting the small number of groups and suggesting modest model efficiency after adjusting for degrees of freedom.

The overall F-statistic was 3.03 (3, 6 degrees of freedom) with a P-value of 0.12, indicating the model was not statistically significant at conventional thresholds. Most importantly, the falsification interaction term (Treat × Placebo Time) was near zero and non-significant (Estimate = –0.0045, P = 0.94), providing strong reassurance that differential trends in early-stage diagnosis rates did not exist prior to policy implementation. This supports the validity of the parallel trends assumption in our primary analysis of stage at diagnosis.

© 2025 Gawdi R et al. *JAMA Network Open*.

eTable 11. Time-Treatment Interaction

Expansion Group	tt() Term	HR	95% CI	p-value
Early Expansion Interaction	Time-Varying Interaction	0.99	0.95–1.03	0.61
	Time Effect	0.90	0.84–0.97	0.003
	Treatment Effect	0.65	0.60–0.71	<0.001
On-Time Expansion Interaction	Time-Varying Interaction	0.98	0.94–1.03	0.48
	Time Effect	0.89	0.83–0.95	<0.001
	Treatment Effect	0.86	0.80–0.92	<0.001
Late Expansion Interaction	Time-Varying Interaction	0.99	0.93–1.05	0.68
	Time Effect	0.85	0.77–0.93	<0.001
	Treatment Effect	1.12	1.05–1.20	<0.001

eTable 11 shows time-dependent Cox regression models that were used to assess whether the effect of Medicaid expansion on mortality varied over follow-up time. tt() indicates the time-varying interaction between follow-up and Medicaid expansion. Across all three models (early, intermediate, and late post-expansion periods), the time-varying interaction term was not statistically significant, indicating no meaningful variation of the hazard over time. Main effects for post-period and treatment remained consistent with the primary Cox model, supporting its robustness.

© 2025 Gawdi R et al. *JAMA Network Open*.

eTable 12. Royston-Parmar Parametric Model

Term	HR	95% CI Lower	95% CI Upper	p-value
Early Expansion: Expansion Status/Time Interaction	0.95	0.85	1.07	0.4
On-Time Expansion: Expansion Status/Time Interaction	0.91	0.81	1.01	0.09
Late Expansion: Expansion Status/Time Interaction	0.95	0.81	1.1	0.49

eTable 12 presents a sensitivity analysis using a Royston-Parmar flexible parametric survival model with 5 degrees of freedom (k=5). The primary interaction terms for all three models (Early, On-Time, and Late Expansion) were not statistically significant (P>0.05). However, the hazard ratios (0.95, 0.91, and 0.95, respectively) were consistently below 1.0 and exactly align with the HR of 2 year survival Cox Models (0.95, 0.91. 0.95), and the confidence intervals were all close to the null value, reinforcing that the proportional hazards assumption is not a major source of bias.

© 2025 Gawdi R et al. *JAMA Network Open*.

eReferences

1. Status of State Medicaid Expansion Decisions. KFF. February 12, 2025. Accessed March 14, 2025. https://www.kff.org/status-of-state-medicaid-expansion-decisions/
2. Enewold L, Parsons H, Zhao L, et al. Updated Overview of the SEER-Medicare Data: Enhanced Content and Applications. *J Natl Cancer Inst Monogr*. 2020;2020(55):3-13. doi:10.1093/jncimonographs/lgz029
3. National Cancer Institute SEER*Stat software. Surveillance Research Program. Published online March 1, 2025. seer.cancer.gov/seerstat
4. Wang J, Trivedi AN. Enrollment in California's Medicaid Program After the Affordable Care Act Expansion. *Am J Public Health*. 2017;107(11):1757-1759. doi:10.2105/AJPH.2017.304031
5. Sommers B, Arntson E, Kenney G, Epstein A. Lessons from Early Medicaid Expansions Under Health Reform: Interviews with Medicaid Officials. *MMRR*. 2013;3(4):E1-E23. doi:10.5600/mmrr.003.04.a02
6. Castro RJ. Repealing the Medicaid Expansion Would Reverse Health Coverage Gains & Deepen New Jersey's Financial Crisis. New Jersey Policy Perspective. November 28, 2016. Accessed May 15, 2025. https://www.njpp.org/publications/report/repealing-the-medicaid-expansion-would-reverse-health-coverage-gains-deepen-new-jerseys-financial-crisis/
7. Sommers BD, Kenney GM, Epstein AM. New evidence on the Affordable Care Act: coverage impacts of early medicaid expansions. *Health Aff (Millwood)*. 2014;33(1):78-87. doi:10.1377/hlthaff.2013.1087
8. Department of Human Services | The Department of Human Services and the Affordable Care Act. Accessed May 15, 2025. https://humanservices.hawaii.gov/aca/
9. The Impact Of State Policies On ACA Applications And Enrollment Among Low-Income Adults In Arkansas, Kentucky, And Texas. Accessed May 15, 2025. https://dash.harvard.edu/entities/publication/73120379-13c4-6bd4-e053-0100007fdf3b
10. Iowa | advancingstates.org. Accessed May 15, 2025. https://www.advancingstates.org/initiatives/tracking-state-activity/state-medicaid-integration-tracker/iowa
11. Filtered Medicaid Enrollment - New Adult Group. Accessed May 15, 2025. https://data.medicaid.gov/dataset/6c114b2c-cb83-559b-832f-4d8b06d6c1b9/data
12. Dunleavy M, Hedberg H. Long Term Forecast of Medicaid Enrollmenta nd Spending in Alaska: FY2025-2045. Published online February 21, 2025. https://health.alaska.gov/media/ya5hhm30/long-term-forecast-of-medicaid-enrollment-and-spending-in-alaska-fy2025fy2045.pdf
13. Medicaid Renewals Data Reporting | Louisiana Department of Health. Accessed May 15, 2025. https://ldh.la.gov/page/medicaid-renewals-data-reporting

© 2025 Gawdi R et al. *JAMA Network Open*.
