# Supplementary material for: Statewide Medicaid Expansion and Survival in Resectable Non–Small Cell Lung Cancer
Source: JAMA Netw Open. 2025 Dec 1;8(12):e2545996. doi: 10.1001/jamanetworkopen.2025.45996 (PMC12670197; doi:10.1001/jamanetworkopen.2025.45996)
Supplement: Supplement 2. — Data Sharing Statement [file jamanetwopen-e2545996-s002.pdf]

Data Sharing Statement

Data
Data available: Yes
Data types: Deidentified participant data, Data dictionary
How to access data: All data used in this study were obtained from the Surveillance, Epidemiology, and End Results (SEER) Research Plus database (version 8.4.4), which is a publicly available deidentified dataset. The data can be accessed by researchers through application to the National Cancer Institute (NCI) and is subject to a data use agreement. The data dictionary is also publicly available from the SEER website. The analytic code used to perform the statistical analyses for this study will be made available upon reasonable request to the corresponding author.
When available: With publication

Supporting Documents
Document types: Statistical/analytic code
How to access documents: https://github.com/rogawdi/NSCLC-Medicaid-Expansion/
When available: With publication

Additional Information
Who can access the data: The analytic code used to perform the statistical analyses is publicly available on a GitHub repository. The link to this repository will be provided upon publication.
Types of analyses: For any research or replication purpose.
Mechanisms of data availability: After approval of a proposal and with a signed data access agreement.
